# Regional Ischemic Preconditioning Has Clinical Value in Cirrhotic HCC Through MAPK Pathways

**DOI:** 10.1007/s11605-018-3960-1

**Published:** 2018-12-12

**Authors:** Liming Wang, Li Feng, Weiqi Rong, Mei Liu, Fan Wu, Weibo Yu, Songlin An, Xiang Zhou, Jianxiong Wu

**Affiliations:** 1 Department of Hepatobiliary Surgery, National Cancer Center/National Clinical Research Center for Cancer/Cancer Hospital, Chinese Academy of Medical Sciences and Peking Union Medical College, No.17 Panjiayuannanli Road, Chaoyang District, Beijing, 100021 China; 2 Department of Ultrasound, National Cancer Center/National Clinical Research Center for Cancer/Cancer Hospital, Chinese Academy of Medical Sciences and Peking Union Medical College, No.17 Panjiayuannanli Road, Chaoyang District, Beijing, 100021 China; 3 Laboratory of Cell and Molecular Biology & State Key Laboratory of Molecular Oncology, National Cancer Center/National Clinical Research Center for Cancer/Cancer Hospital, Chinese Academy of Medical Sciences and Peking Union Medical College, Beijing, 100021 China; 4 Department of Pathology and Laboratory Medicine, University of California, Los Angeles, CA USA; 5 Department of Peritonea Cancer Surgery, Beijing Shijitan Hospital, the 9th Clinical Medical College of Peking University and Clinical Cancer Center of Capital Medical University, Beijing, China

**Keywords:** Hepatocellular carcinoma, Cirrhosis, Regional ischemic preconditioning, Mitogen-activated protein kinase

## Abstract

**Background:**

This study assessed the clinical value of regional ischemic preconditioning (RIP) and the role of the mitogen-activated protein kinase (MAPK) pathways in the protective mechanism of RIP in cirrhotic hepatocellular carcinoma (HCC) patients undergoing hepatectomy.

**Methods:**

Liver resection was performed with hemi-hepatic vascular inflow occlusion (HHV) under RIP (RIP group) or with HHV alone (HHV group). Clinical data, surgical outcomes, and the levels of phosphorylated MAPKs before occlusion and 30 min after reperfusion were estimated.

**Results:**

HHV under RIP was associated with less intraoperative blood loss (300 vs. 400 ml; *P* = 0.042), postoperative plasma transfused (400 vs. 800 ml; *P* = 0.019), and a higher level of prothrombin activity at postoperative days 3, 5, and 7 compared to HHV alone. The level of phosphorylated ERK protein was significantly increased and the levels of phosphorylated p38 and JNK proteins were significantly decreased 30 min after reperfusion compared to HHV group in the RIP group.

**Conclusions:**

HHV under RIP may have clinical value in cirrhotic HCC patients requiring resection and the protective mechanism of RIP may be associated with changes in the protein phosphorylation level of MAPK pathways.

**Electronic supplementary material:**

The online version of this article (10.1007/s11605-018-3960-1) contains supplementary material, which is available to authorized users.

## Introduction

Malignant liver tumors, including hepatocellular carcinoma (HCC), are treated by elective liver resection. Blood loss during liver resection is prevented by vascular occlusion, which can result in ischemia/reperfusion (IR) injury. It is generally accepted that the cirrhotic liver is particularly sensitive to IR injury.^1,2^

Worldwide, liver cancer is the fifth most frequently diagnosed cancer, accounting for more than 700,000 deaths per year. In 2008, almost 55% of global liver cancer cases and deaths occurred in China.^3^ In China and other Asian countries, more than 80% of HCC patients have underlying liver cirrhosis due to hepatitis B virus infection.^4,5^ Therefore, the need for effective methods to reduce the IR injury associated with vascular occlusion during liver resection in cirrhotic HCC patients has become increasingly important.

Currently, ischemic preconditioning (IP) is recommended to protect against hepatic IR injury. However, most studies supporting this approach focused on patients with benign tumors or HCC without liver cirrhosis, which are not applicable to Asian countries, and outcomes of human studies on IP during hepatectomy are controversial. Some studies have confirmed the protective effect of IP. In a randomized controlled trial, patients undergoing hemihepatectomy under IP (10 min ischemia/10 min reperfusion) had significantly lower levels of markers of hepatic injury (aspartate transferase [AST], alanine transferase [ALT]) during the immediate postoperative period than controls.^6^ In patients who received hepatic resection under IP, intraoperative bleeding and postoperative complications were significantly reduced compared to no IP,^7^ and blood transfusions.^8^ hepatocyte injury, and duration of surgery^9^ were decreased compared to alternative methods of vascular occlusion. Other studies showed that IP (10 min ischemia/10 min reperfusion) had no clinical value for postoperative liver function and reducing postoperative complications and mortality in major liver resection,^10^ and there were no significant differences in operative time, blood loss, hemodynamic changes, postoperative liver function, postoperative complications, and length of hospital stay in patients undergoing elective liver resection under the Pringle maneuver^11,12^ or complex hepatectomy under total vascular exclusion (TVE) with or without IP.^13^

Most surgeons use the Pringle maneuver to reduce blood loss during liver resection. However, recent reports suggest that the Pringle maneuver is associated with portal vein thrombosis, spontaneous rupture,^14^ IR injury^15^ and may accelerate tumor recurrence.^16^ Hemi-hepatic vascular inflow occlusion (HHV) is increasingly used as an alternative to the Pringle maneuver.^17,18^ HHV keeps the contralateral hepatic blood supply open, ameliorates IR injury, contributes to portal hemodynamic stability, prevents intestinal mucosal damage induced by mesenteric vascular congestion, and achieved earlier postoperative recovery of liver function compared to the Pringle maneuver in a randomized controlled trial of patients undergoing partial hepatectomy.^19^

Accounting for the controversy surrounding IP and the advantages of HHV, we propose the use of regional ischemic preconditioning (RIP) in cirrhotic HCC patients. This process involves occluding only 50% of the hepatic blood inflow. Data from our preliminary clinical trial indicated that HHV under RIP may be safer and achieve earlier postoperative recovery of liver function than HHV alone in HCC patients.^20^

The protective mechanism of RIP remains to be elucidated. The pathophysiology of hepatic IR may include a number of mechanisms that the formation of pro- and anti-inflammatory mediators, expression of adhesion molecules, and the role of oxidant stress, preventing microcirculatory disturbances by nitric oxides.^21^ Recent studies have identified mitogen-activated protein kinase (MAPK) as the major intracellular signal transduction system in IR injury.^22^ MAPKs are regulated by phosphorylation and have key roles in the control of cell proliferation, differentiation, and apoptosis.^23^ In mammalian cells, the MAPK cascade includes the ERK pathway, JNK pathway, and the p38 pathway. ERK is an important transcription factor, and the JNK and p38 pathways are involved in cellular responses to environmental stress and inflammatory cytokines. IR injury, heat shock, hypertonic conditions, and certain inflammatory cytokines such as tumor necrosis factor can activate the MAPK signaling pathway. MAPKs are involved in IR injury;^24,25^ however, the most of the previous researches were performed in cell or animal models, clinical studies in hepatic IR injury and those considering IP are limited.

The objective of the current study was to conduct a prospective non-randomized controlled trial comparing HHV under RIP with HHV alone in cirrhotic HCC patients undergoing liver resection to assess the clinical value of RIP and explore the role of the MAPK pathways in the endogenous protective mechanism of RIP in these patients.

## Materials and Methods

### Patients and Tissue Samples

The study protocol was approved by the Institutional Review Board at the Cancer Hospital, Chinese Academy of Medical Sciences. A total of 91 cirrhotic patients requiring resection for HCC were eligible for this study. Inclusion criteria were as follows: ≥ 18 years of age; primary HCC diagnosed by imaging and serum alpha-fetoprotein (AFP); compensated cirrhosis (Child-Pugh score grade A); tumor located in the right lobe of liver, close to the right hepatic vein, right portal vein, inferior vena cava or other important blood vessels, or not near major blood vessels but with a diameter > 5 cm; single or multiple lesions confined to three segments; laparotomy; indocyanine Green Retention rate at 15 min (ICG-R15) < 15%; and absence of serious cardiopulmonary complications. Exclusion criteria were as follows: other liver pathology or previous interventions (e.g., radiation therapy, radiofrequency therapy).

As a non-randomized cohort research, patients were assigned to a RIP group (HHV under RIP) or an HHV group (HHV alone) alternately with a ratio of 1:2. Case number was calculated by software PASS according to previous research. All patients consulted with a research nurse, who provided patients with comprehensive information about the advantages and disadvantages of the two occlusion techniques. All patients provided written informed consent before participation in the study.

Specimens of liver tissue (wedge, approximately 0.5–0.8 cm^3^) were obtained from subjects before occlusion and 30 min after reperfusion. Each specimen was divided into two parts. One part was immediately placed in liquid nitrogen and stored at −80 °C until Western blotting. The other part was washed three times in PBS buffer, fixed in 10% neutral formalin solution, dehydrated, embedded in paraffin, and sectioned for H&E staining and immunohistochemistry.

### Surgical Procedure

All operations were performed by one board-certified hepatobiliary surgeon with a single, dedicated surgical support team. After laparotomy, the abdominal and pelvic areas were carefully explored to determine the location, size, and number of liver tumors, tumor location relative to important hepatic ducts and vessels, and resection extent. Intraoperative ultrasound was used if necessary.

In the HHV group, liver resection was performed with HHV only. An ultrasonic scalpel or electrotome with excellent hemostatic effect was used to relatively slowly dissect the liver surface. Continuous HHV was performed while dissecting deep inside the liver tissue. Vascular anastomosis silk was used to ligate or suture the cut surface of the liver when bleeding was obvious. After complete resection, intrahepatic structures (blood vessels and bile ducts) and the liver surface were repaired, and the continuous hemi-hepatic inflow occlusion was de-clamped. The natural position of the remnant liver was retained after covering the cut surface with a hemostatic sponge.

**Fig. 1:**
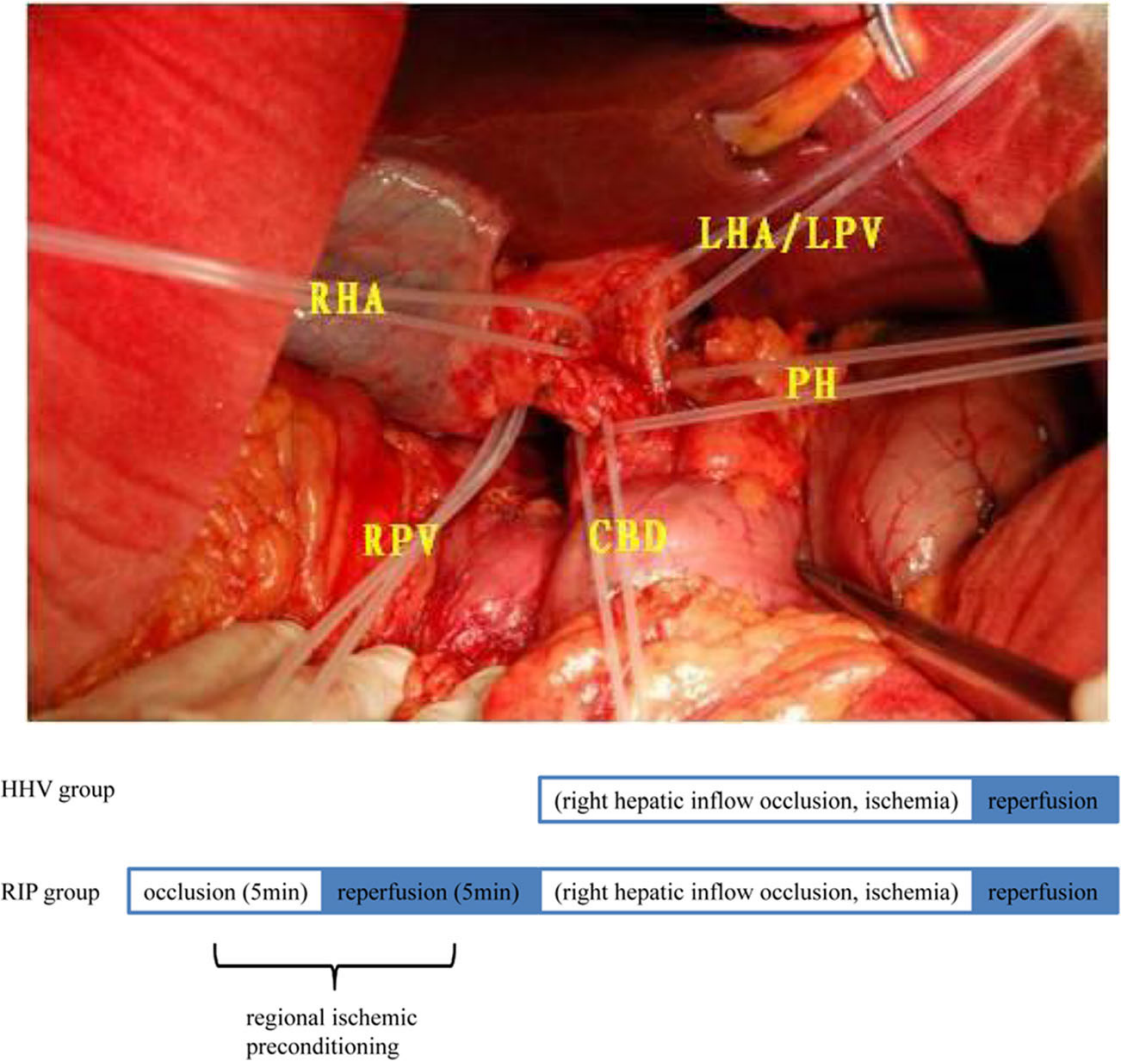
Treatment schemes. The first porta hepatis was elaborately dissected to reveal the left and right branch of the hepatic artery and portal vein; a rubber band was preset on each vessel. The right hepatic inflow was occluded by tightening the rubber band on the right branch of the hepatic artery (RHA) and portal vein (RPV); the occluded right hepatic inflow was restored by loosening the rubber band. Methodology of regional ischemic preconditioning is based on right hepatic inflow occlusion. RHA, right hepatic artery; RPV, right portal vein; PH, porta hepatis; CB, common bile duct; LHA/LPV, left hepatic artery/left portal vein

In the RIP group, liver resection was performed with HHV after a short period of RIP. According to previous studies, 5 min ischemia/5 min reperfusion is recommended for IP in the cirrhotic liver.^26^ The first porta hepatis was elaborately dissected to reveal the left and right branch of the hepatic artery and portal vein; a rubber band was preset on each vessel (Fig. 1). Clamping of the hemi-portal inflow was performed with the tourniquet technique using rubber tape. The right hepatic inflow was occluded by tightening the rubber band on the right branch of the hepatic artery and portal vein. Immediately after a brief period of ischemia (5 min) followed by 5 min of reperfusion (RIP), liver transection was initiated under continuous HHV as HHV group.

Postoperative complications were evaluated according to clinical practice and published literature.^27–29^

### Immunohistochemistry

Immunohistochemistry was performed as previously described.^30^ Primary antibodies were used including ERK (1:100, Cell Signaling Technology), phosphorylated ERK (1:100, Cell Signaling Technology), p38 (1:100, Cell Signaling Technology), phosphorylated p38 (1:100, Cell Signaling Technology), JNK (1:50, Santa Cruz Biotechnology), and phosphorylated JNK (1:50, Cell Signaling Technology). Sections were counterstained with Harris’ hematoxylin. Slides were scored according to staining intensity and the proportion of positive cells. Staining intensity was determined according to the staining characteristics and was scored as follows: no staining, 0 (None); light yellow, 1 (Weak); brown yellow, 2 (Moderate); brown, 3 (Strong). Five fields were randomly selected at a high magnification and 100 cells were counted in each field in which the proportion of positive cells was determined and scored as follows: 1 = 0 to 10%; 2 = 11–25%; 3 = 26–50%; 4 = > 50%. The total score was the multiplication of both scores: 0, negative (−), 1 to 4, weakly positive [+]; 5–8, moderately positive [++]; 9–12, strongly positive [+++]. All the sections were scored independently by two experienced pathologists.

### Western Blot Analysis

Western blot analysis was performed with the use of conventional protocols as described previously^31^ using liver homogenates and the above-mentioned antibodies. All the antibodies (including GAPDH (Proteintech)) were used at 1:1000 dilution. Densitometry was performed using ImageQuant LAS 4000 mini (GEHealthcare). Data was analyzed with Image J software.

### Statistical Analysis

Statistical analysis was performed using SPSS software version 16.0 (SPSS, Inc., Chicago, IL). Continuous data are expressed as mean ± standard deviation and were compared using an independent Student’s *t* test or Wilcoxon rank sum test. Categorical data were compared using the *χ*^2^ test or Fisher’s exact probability test. *P* < 0.05 was considered statistically significant.

## Results

### Study Subjects

A total of 91 patients were eligible for inclusion in this study. Of these, 15 patients did not meet the inclusion criteria, and 4 patients refused to participate; therefore, 72 patients were included in the analyses. A total of 23 patients chose to be included in the RIP group and 49 patients chose to be included in the HHV group. Demographic and clinical characteristics of the study subjects are shown in Table 1. Mean age was 55.4 years (range, 31–72 years) and mean tumor diameter was 4.4 cm (1.0–14.5 cm). Thirty-four tumors were > 5 cm in diameter and 41 tumors were located close to large vessels. There were no significant differences in demographic and clinical characteristics between the two groups.

**Table 1:** Clinicopathological characteristics of the patients

Characteristics	RIP group (*n* = 23)	HHV group (*n* = 49)	*P* value
Age (years, mean ± SD)	56.5 ± 10.3	55.0 ± 8.9	0.535
Sex (male/female, *n*)	19/4	36/3	0.556
Hepatitis B surface antigen serology positive (*n*)	16	38	0.466
Hepatitis C surface antigen serology positive (*n*)	3	3	0.322
Cirrhosis (*n*)			0.994
Mild/moderate	15	32	
Severe	8	17	
Size of tumor (cm, median and range)	4.5 (2.0–14.5)	4.4 (1.0–9.5)	0.184
Number of tumor (*n*)			0.429
Single	18	42	
Multiple	5	7	
Vascular involvement (*n*)			0.939
RHV	5	10	
RPV	2	1	
MHV	1	2	
MHV and RPV	1	2	
RHV and RPV	1	3	
MHV and RHV	2	1	
IVC and RHV	1	2	
Adhesion to the third hepatic hilum	1	3	
IVC and RHV and RPV	1	2	

Vascular involvement was defined as tumor close to venous wall less than 1 cm or resectable attachment of HCC onto the venous wall

*RIP* regional ischemic preconditioning, *HHV* hemi-hepatic vascular inflow occlusion, *RHV* right hepatic vein, *RPV* right portal vein, *MHV* middle hepatic vein, *IVC* inferior vena cava

### Intraoperative and Postoperative Outcomes

Intraoperative and postoperative outcomes are shown in Table 2. There were no significant differences in operative procedure, liver resection volume, incisal margin, operative time, continuous regional ischemia time, and number of intraoperative blood transfusions between the two groups.

**Table 2:** Operative outcomes

Characteristics	RIP group (*n* = 23)	HHV group (*n* = 49)	*P* value
Operative procedure (major/minor)^a^	10/13	21/28	0.960
Resection volumes (cm^3^)	288	264	0.740
Incisal margin (*n*)			0.445
0 cm	13	20	
0–2 cm	2	7	
≥ 2 cm	8	22	
Intraoperative bile duct damage (*n*)	1	4	1.000
Duration of surgery (min, mean ± SD)	222.4 ± 69.4	236.4 ± 60.2	0.383
Continuous regional ischemia time (min)	21(10–58)	18(10–70)	0.272
Intraoperative bleeding (ml, median and range)	300 (100–1100)	400 (100–1500)	*0.042*
Total intraoperative blood transfused (ml, median and range)	900 (600–1200)	1200(400–2400)	0.445
Intraoperative transfusions (cases)	2	7	0.504
Postoperative plasma transfused (ml, median and range)	400 (200–400)	800 (400–2800)	*0.019*
Postoperative RBC transfused (cases)	0	4	0.299
Postoperative plasma transfused (cases)	4	17	0.132
ALT recovering within 1 week (*n*)	8	1	*< 0.001*
Postoperative hospital stay (*n*, median and range)	9 (2–17)	9 (6–49)	0.932
Complications (*n*)			
Hepatic dysfunction	1	5	0.657
Mass ascites	1	8	0.260
Postoperative infection	2	10	0.214
Pleural effusion	0	3	0.547
Pulmonary and cardiac dysfunction	0	5	0.170
Bile leak	0	0	–
Intestinal ventilation time	4 (2–6)	4 (2–6)	0.311

Italic emphasis: *P* value is less than 0.05

*RIP* regional ischemic preconditioning, *HHV* hemi-hepatic vascular inflow occlusion, *ALT* alanine aminotransferase, *RBC* red blood cell

Operative procedure^a^: Minor resection: no more than one segment resection;Major resection: two or three segments resection (more than one segment and less than hemihepatectomy).

There was less intraoperative blood loss (300 vs. 400 ml; *P* = 0.042) and postoperative plasma transfused (400 vs. 800 ml; *P* = 0.019), in the RIP group compared to the HHV group. There were no deaths or biliary fistulas in either group at postoperative 30 days. There were no significant differences in postoperative length of hospital stay, postoperative infection, pleural effusion, and cardiac and pulmonary dysfunction between the two groups.

### Liver Function Tests After Liver Resection

**Fig. 2:**
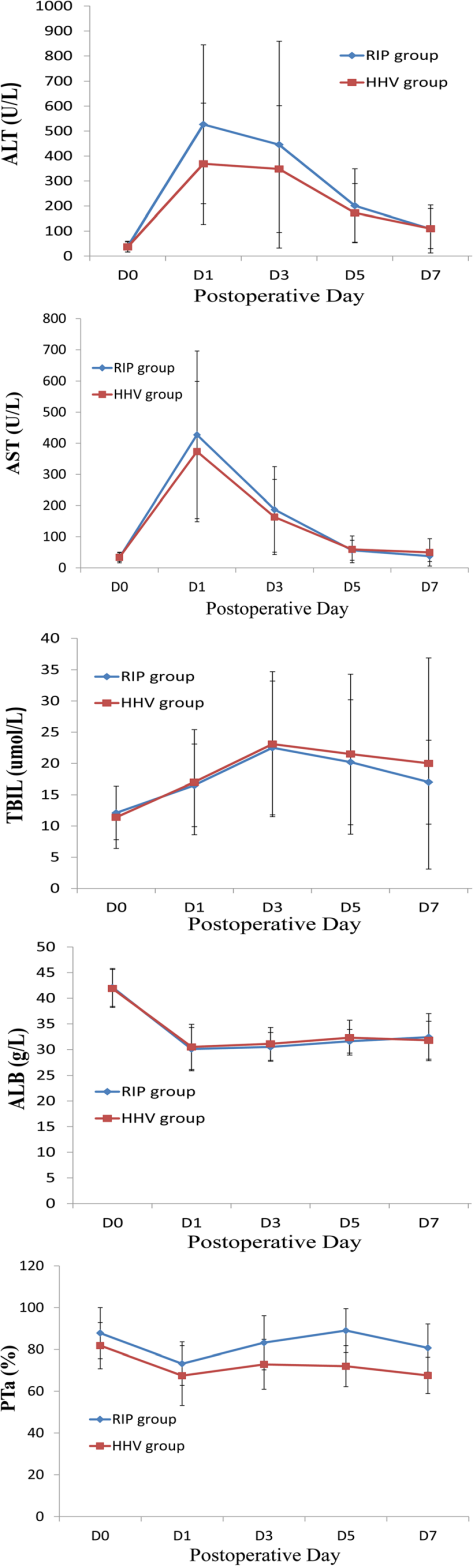
Liver function tests after liver resection with (blue line, *n* = 23) versus without (red line, *n* = 49) RIP. There were no significant differences between the RIP and HHV groups except for prothrombin activity at postoperative days 3, 5, and 7 (*P* < 0.05). D0, preoperative day; D1, postoperative day 1; D3, postoperative day 3; D5, postoperative day 5; D7, postoperative day 7; ALT, alanine transaminase; AST, aspartate transaminase; TBIL, total bilirubin; ALB, albumin; PTA, prothrombin activity

Outcomes of liver function tests after liver resection are shown in Table 2 and Fig. 2. PTA was significantly higher on postoperative days 3, 5, and 7 in the RIP group compared to the HHV group (*P* < 0.05) and ALT returned to normal levels within 1 week in significantly more patients in the RIP group than the HHV group (8 vs. 1; *P* < 0.001). There were no differences in postoperative AST, ALT, ALB, or TBIL levels between the two groups in each detection point (*P* > 0.05).

In both groups, AST and ALT levels increased to a maximum and ALB levels decreased to a minimum on postoperative day 1; TBIL levels increased to a maximum on postoperative day 3. The trend in changes in AST, ALT, ALB, and TBIL levels was similar in both groups and not significantly different. PTA decreased on postoperative day 1 and then rose; there was a significant difference in PTA between the two groups on postoperative day 5 (RIP, 89%; HHV, 72%; *P* < 0.001) (Fig. 2).

### Histopathology

**Fig. 3:**
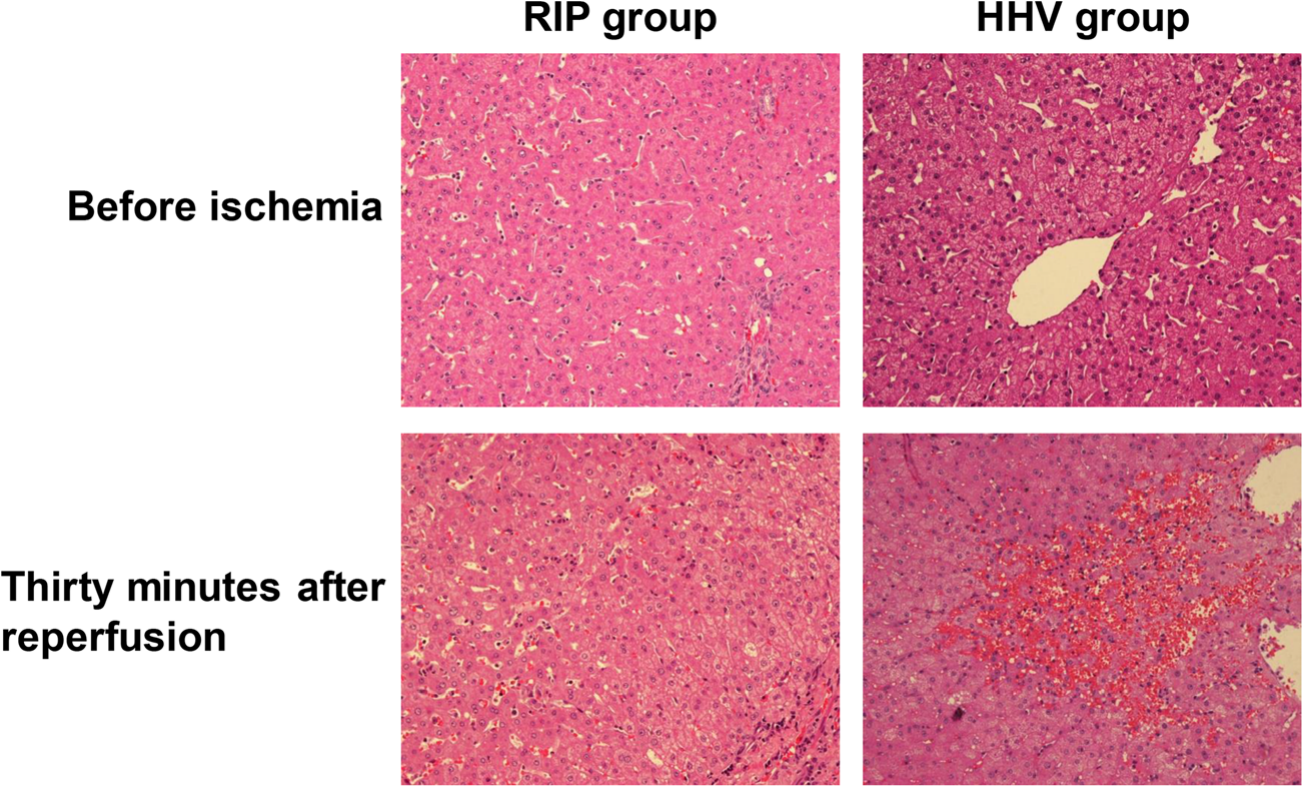
Liver morphology change before vascular occlusion and 30 min after reperfusion. The RIP group showed less hepatocyte injury and inflammatory cell infiltration than the HHV group. ×200. HHV, hemi-hepatic vascular inflow occlusion; RIP, regional ischemic preconditioning

There were no histopathological differences between the two groups before liver resection. Thirty minutes after reperfusion, in the HHV group, there were enlarged hepatocytes, centrolobular venous dilation and congestion, sinusoidal congestion, an obvious inflammatory cell infiltration, and incomplete liver tissue. In the RIP group, there was reduced structural damage compared to the HHV group, with partial ballooning degeneration of hepatocytes, no sheet cell necrosis, and less sinusoidal congestion and inflammatory cell infiltration (Fig. 3).

## pJNK, pp38, and pERK Proteins Levels in the RIP Group and HHV Group

**Fig. 4:**
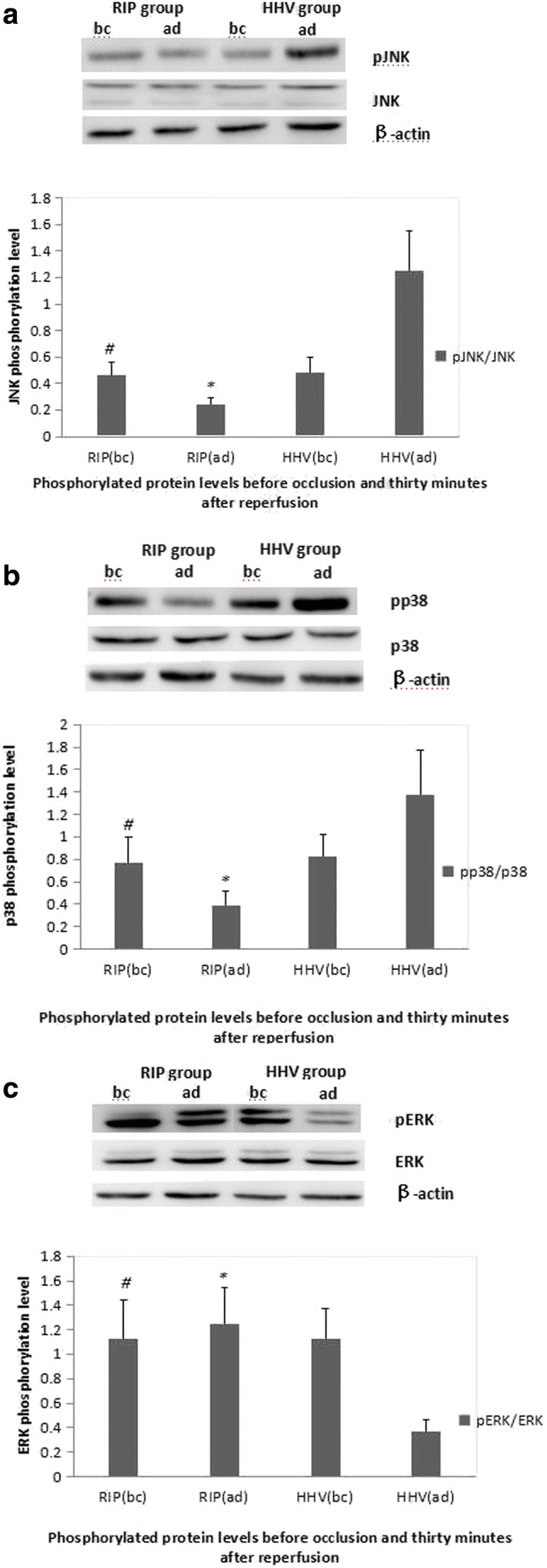
Protein phosphorylation levels before vascular occlusion and 30 min after reperfusion. **a** JNK phosphorylation level; **b** p38 phosphorylation level; **c** ERK phosphorylation level. bc, before; ad, after; HHV, hemi-hepatic vascular inflow occlusion; RIP, regional ischemic preconditioning; p, phosphorylated. The level of pERK was increased and the levels of pJNK and pp38 were decreased in the RIP group 30 min after reperfusion compared to the HHV group. # compared to HHV group before ischemia *P* > 0.05, *compared to HHV group after occlusion *P* < 0.05

As shown in supplementary Figure 1, there were no significant differences in total JNK, p38, and ERK protein levels between the two groups. However, phosphorylated JNK and phosphorylated p38 protein levels were decreased in the RIP group and increased in the HHV group 30 min after reperfusion compared to before occlusion (*P* < 0.05) (Fig. 4a, b). The level of phosphorylated ERK protein was increased in the RIP group and decreased in the HHV group 30 min after reperfusion compared to before occlusion. Thirty minutes after reperfusion, the level of phosphorylated ERK protein was significantly higher in the RIP group compared to the HHV group (*P* < 0.05) (Fig. 4c).

Immunohistochemistry staining showed the similar results. In all tissues, phosphorylated ERK and phosphorylated p38 proteins were localized to the sinusoidal endothelial cells, and phosphorylated JNK protein was localized in the hepatocytes. Before occlusion, there were no significant differences in the levels of phosphorylated JNK, phosphorylated p38, and phosphorylated ERK proteins between the two groups (data not shown). Thirty minutes after reperfusion, the levels of phosphorylated JNK and phosphorylated p38 proteins were significantly lower in the RIP group than the HHV group, while the level of phosphorylated ERK protein was significantly higher in the RIP group (*P* < 0.001; Table 3) (Supplementary Fig. 1).

**Table 3:** Phosphorylated protein levels before occlusion and 30 min after reperfusion

	Group	+ (cases)	++ (cases)	+++ (cases)	*P* value^*^
Before occlusion					
pJNK	HHV	27	17	5	
	RIP	12	9	2	0.929
pp38	HHV	42	7	0	
	RIP	20	3	0	1.000
pERK	HHV	12	28	9	
	RIP	9	8	6	0.206
Thirty minutes after reperfusion					
pJNK	HHV	2	31	16	
	RIP	14	5	4	< 0.001
pp38	HHV	13	34	2	
	RIP	18	5	0	< 0.001
pERK	HHV	25	21	3	
	RIP	3	8	12	< 0.001

*HHV* hemi-hepatic vascular inflow occlusion, *RIP* regional ischemic preconditioning, *pJNK* phosphorylated JNK, *pp38* phosphorylated p38, *pERK* phosphorylated ERK

^*^Two-sided Person’s *χ*^2^ test or Fisher’s exact test

## Discussion

This prospective non-randomized controlled trial studied the effect of HHV under RIP on cirrhotic HCC patients undergoing liver resection. HHV under RIP was associated with less intraoperative blood loss and less postoperative plasma transfused compared to HHV alone.

These findings suggest that RIP may reduce intraoperative blood loss in cirrhotic HCC patients undergoing liver resection. However, the results should be interpreted with caution as their clinical significance remains to be elucidated. We attribute the reduced intraoperative blood loss in the RIP group compared to the HHV group to the higher intraoperative PTA observed in the RIP group. However, this supposition requires confirmation through dynamic studies that monitor intraoperative PTA in HHV under RIP and HHV alone. In our cohort study, there was no significant difference between the two groups in body mass index (BMI) and resection volumes, respectively, so we speculated that they may be not the main cause for intraoperative bleeding. However, four more TACE-treated patients enrolled in the HHV group compare to RIP group because of unclear reason. Previous study have showed that patients underwent preoperative TACE had more intraoperative blood loss because of perihepatic organs adhesion.^32^ This may be one of the main reasons for blood loss.

We determined the need for postoperative plasma transfusions according to intraoperative exudation in the cirrhotic liver, volume of postoperative blood-tinged fluid, and in particular postoperative PTA (representing coagulation function), in order to determine the amounts of coagulation factors and plasma proteins to replace. There was no significant difference between the two groups in PTA on postoperative day 1, but PTA was significantly lower on postoperative days 3, 5, and 7 in the HHV group compared to the RIP group, which showed that the recovery of PTA in the HHV group was slower than that of the RIP group. This suggests that RIP may have some protective effect on coagulation function, causing patients in the RIP group to have less intraoperative exudation and less postoperative plasma transfused. More importantly, it should be noted that our current study included only patients with HCC and liver cirrhosis. Over 50% of tumors were located near the right or middle hepatic vein, right portal vein, the third porta of the liver, or the inferior vena cava. These were considered high-risk patients who were likely to experience postoperative liver failure. To the authors’ knowledge, the current study is the first to investigate the effects of RIP in cirrhotic HCC patients undergoing liver resection. The outcome of the current study is in accordance with our preliminary study of 54 HCC patients who underwent hepatectomy under HHV with or without RIP^20^ and a randomized controlled trial of 61 patients who received hepatic resection under the Pringle maneuver with or without IP.^7^ In these studies, IP significantly reduced intraoperative bleeding^7,20^ and postoperative complications^7^ and promoted early recovery of liver function^20^ compared to no IP.

For cirrhotic patients, continuous portal triad clamping (Pringle maneuver) exceeding 30 min may cause irreversible injury. Previously, we have shown that the maximum ischemic tolerance time of the liver in HCC patients is 70 min.^20^ This study showed the same maximum tolerance time. Total ischemic time in this study group ranging from 10 to 70 min was determined by difficulty of surgical procedure instead of a fixed time period. There was no significant difference of mean ischemia time between the two groups. But, the changes of molecular and histopathology profile were truly observed in all the patients even the least ischemic time was only 10 min. Although the degree of changes of molecular and histopathology profile theoretically correlated with ischemic time, the detail of the correlation was not the target we focused, this could be an exploratory goals in future. We propose that HHV under RIP can prolong the ischemic tolerance time of the liver and therefore has potential to increase the duration of surgery. This is especially important in patients with severe disease, such as those with cirrhosis or tumors located close to intrahepatic structures. Furthermore, we found that HHV under RIP was associated with faster postoperative recovery of transaminase levels and therefore liver function, and less intraoperative liver tissue damage compared to HHV alone.

The current study showed that the operative time, length of hospital stay, and frequency of postoperative complications were not different between the RIP and HHV groups, indicating that the safety of HHV was not compromised by RIP. These data are in accordance with a prospective randomized controlled study^33^ that used the Pringle maneuver under IP.

IP can mobilize endogenous protective mechanisms and enhance liver tissue tolerance of ischemia. MAPK cascade pathways are involved in cellular responses to extracellular stimuli.^34^ MAPK is a highly conserved family of protein kinases that is regulated by phosphorylation and includes JNK, p38, and ERK. JNK regulates cell viability, apoptosis, and cell proliferation. In the current study, 30 min after reperfusion, the levels of phosphorylated JNK protein and phosphorylated p38 protein were significantly lower in liver tissue from the RIP group compared to the HHV group. These findings are in accordance with a previous study that showed JNK activity was decreased in rat liver after IP, and hepatic injury was alleviated. This suggests that JNK activation may be associated with IR injury.^35^ The role of p38 in tissue damage is controversial. In rats,^36^ p38 activation was associated with increased levels of F-actin in liver cells, changes in the cytoskeleton, and liver failure. ERK is associated with cell proliferation, transformation, and differentiation.^37^ After reperfusion, the level of phosphorylated ERK protein in liver tissue of the RIP group was significantly higher than the HHV group. Studies on ischemia in the myocardium propose that a “RISK path” (reperfusion injury salvage kinases) that includes ERK1/2, PI3K/AKT, PKC, and PKG has a protective effect against IP.^38^ Zhang et al.^39^ reported IP could reduce ischemia/reperfusion-induced cell apoptosis by activating the ERK signaling pathway and inhibiting Rho-kinase activity. Importantly, MAPK pathways are part of a phosphorelay system.^40^ During IP, direct feedback between the ERK and p38 and JNK and p38 pathways is important.^41–43^ p38 and JNK pathways are activated within a few minutes after reperfusion, inducing cell apoptosis and necrosis.^44^ Application of a p38/JNK activator elevates transaminase levels and increases liver necrosis, while a p38/JNK inhibitor may reduce hepatic IR injury.^45^

This study is associated with several limitations. First, patients were not randomly assigned to RIP or HHV; this may result in patient selection and confounding bias. However, demographic and clinical characteristics of the two groups were not significantly different. Second, evidence suggests that the protective effect of IP is age-specific. IP is particularly effective in young patients (< 60 years of age) requiring a prolonged period of inflow occlusion, and is possibly related to preservation of ATP content in liver tissue.^33^ In the current study, patients were not stratified by age due to a small sample size. Third, recovery of transaminase and molecular profiles seems different according to the use of ischemic preconditioning. However, there was no difference in short-term clinical outcomes between the two groups. The current results suggest that preconditioning may have protective effect for the liver from ischemic injury. However, it remains difficult to determine the necessity of this maneuver in actual clinical settings.

## Conclusions

Our study indicates that HHV under RIP may reduce intraoperative IR injury, improve coagulation. and increase the rate of recovery of postoperative liver function compared with HHV alone in HCC patients with cirrhosis requiring resection. The protective mechanism of RIP may be associated with changes in the protein phosphorylation level of MAPK pathways.

## Electronic Supplementary Material

Supplementary Fig. 1 Representative cases of immunohistochemistry staining for pJNK, pp38, and pERK in RIP group and HHV group are shown. Magnification: ×100. (PNG 819 kb)

High resolution image (TIF 13752 kb)

## Abbreviations

AFP: Alpha-fetoprotein
ALB: Albumin
ALT: Alanine transferase
AST: Aspartate transferase
DAB: 3,3′-Diaminobenzidine tetrachloride
HCC: Hepatocellular carcinoma
H&E: Hematoxylin eosin staining
HHV: Hemi-hepatic vascular inflow occlusion
IR: Ischemia/reperfusion
IP: Ischemic preconditioning
MAPK: Mitogen-activated protein kinase
PTA: Prothrombin activity
RIP: Regional ischemic preconditioning
TBIL: Total bilirubin
TVE: Total vascular exclusion
