# Supplementary figures and images for: Regional Ischemic Preconditioning Has Clinical Value in Cirrhotic HCC Through MAPK Pathways
Source: J Gastrointest Surg. 2018 Dec 12;23(9):1767–77. doi: 10.1007/s11605-018-3960-1 (PMC6702190; doi:10.1007/s11605-018-3960-1)

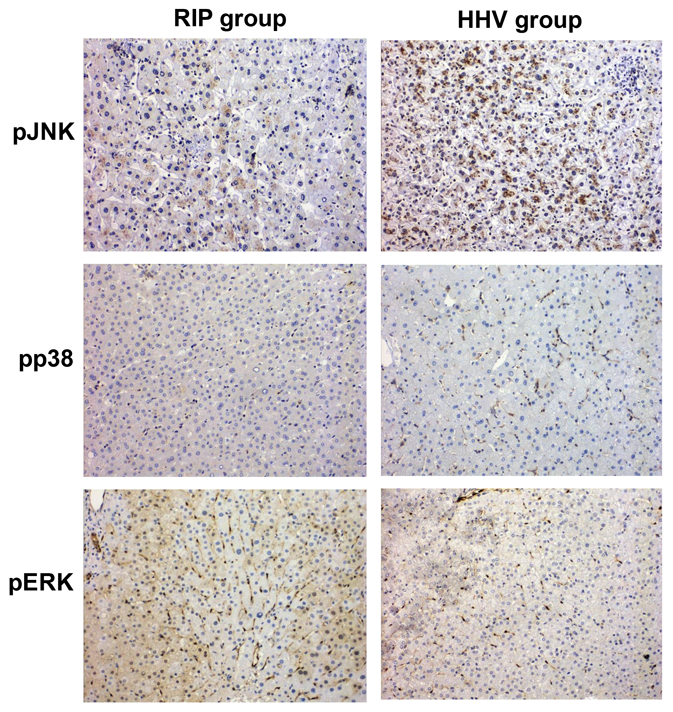

Supplement: Supplementary file 1 — Representative cases of immunohistochemistry staining for pJNK, pp38, and pERK in RIP group and HHV group are shown. Magnification: ×100. (PNG 819 kb) [file 11605_2018_3960_Fig4_ESM.png]

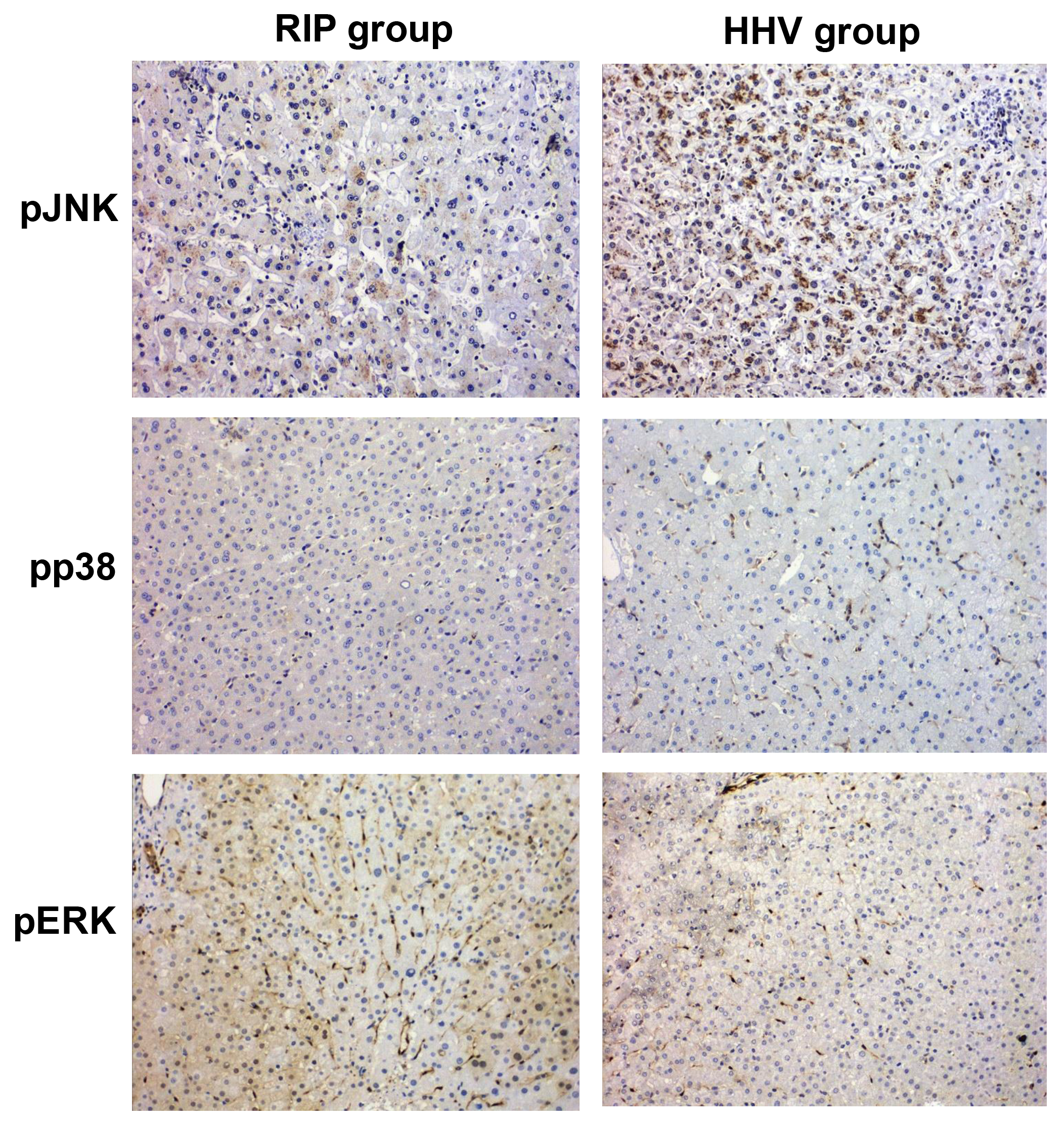

Supplement: Supplementary file 2 — High resolution image (TIF 13752 kb) [file 11605_2018_3960_MOESM1_ESM.tif]
